# The Right Ventricle—You May Forget It, But It Will Not Forget You

**DOI:** 10.3390/jcm9020432

**Published:** 2020-02-05

**Authors:** Patrick M. Wanner, Miodrag Filipovic

**Affiliations:** 1 Department for Anesthesia, Surgical Intensive Care, Prehospital Emergency Medicine and Pain Therapy, University Hospital Basel, 4031 Basel, Switzerland; 2 Division of Anesthesiology, Intensive Care, Rescue and Pain Medicine, Kantonsspital St. Gallen, 9007 St. Gallen, Switzerland

**Keywords:** right ventricle, right ventricular, dysfunction, failure, shock, perioperative, postoperative, management, prevention

## Abstract

Right ventricular (RV) dysfunction and failure are common and often overlooked causes of perioperative deterioration and adverse outcomes. Due to its unique pathophysiologic underpinnings, RV failure often does not respond to typical therapeutic measures such as volume resuscitation and often worsens when therapy is escalated and mechanical ventilation is begun, with a danger of irreversible cardiovascular collapse and death. The single most important factor in improving outcomes in the context of RV failure is anticipating and recognizing it. Once established, a vicious circle of systemic hypotension, and RV ischemia and dilation is set in motion, rapidly spiraling down into a state of shock culminating in multi-organ failure and ultimately death. Therapy of RV failure must focus on rapidly reestablishing RV coronary perfusion, lowering pulmonary vascular resistance and optimizing volemia. In parallel, underlying reversible causes should be sought and if possible treated. In all stages of diagnostics and therapy, echocardiography plays a central role. In severe cases of RV dysfunction there remains a role for the use of the pulmonary artery catheter. When these mostly simple measures are undertaken in a timely fashion, the spiral of death of RV failure can often be broken or even prevented altogether.

## 1. Introduction

Right ventricular (RV) failure is a common, but yet underrecognized cause of perioperative morbidity and mortality. In various disease entities (e.g., severe aortic valve stenosis [1], acute respiratory distress syndrome [ARDS] [2], pulmonary embolism [3]) RV dysfunction portends a marked worsening of prognosis. This also holds true in the perioperative setting with pulmonary hypertension an important predictor of adverse outcomes in noncardiac surgery [4,5]. Additionally, relevant right ventricular dysfunction is found in up to 25% of hemodynamically unstable post-operative patients and its diagnosis leads to a change in management in 59% of cases [6]. Finally, the timely diagnosis of RV failure is paramount due to the fundamentally different treatment approaches in comparison to other forms of acute heart failure, but also due to the dismal prognosis if the diagnosis is missed. In this review, we will summarize the most important points of the perioperative management of patients at risk of or with established RV failure in noncardiac surgery.

## 2. Key Pathophysiology of Right Ventricular Failure

The right ventricle (RV) has numerous anatomical and physiological features that distinguish it from the left ventricle (LV) and which have direct consequences on the diagnostic and therapeutic approach in states of RV dysfunction. In this review, we focus on the key concepts necessary for understanding the core elements of diagnosing and treating RV failure in the perioperative setting. For an in-depth review of RV anatomy and physiology, the reader is encouraged to consult the excellent reviews of Haddad et al. [7,8].

### 2.1. The Right Ventricle is Volume-Tolerant and Pressure-Intolerant

The RV and LV have fundamentally different roles in the circulation. The LV is primarily focused on guaranteeing adequate systemic and coronary perfusion, whereas the RV’s role is to match systemic venous return to LV function and to ensure optimal pulmonary perfusion for gas exchange.

In contrast to the systemic circulation the pulmonary circulation generally has a low resistance vascular bed. This has the consequence that the RV is optimized for low afterload conditions. Although the RV easily handles varying amounts of preload, it rapidly decompensates when faced with an acute rise in afterload, e.g., with an acute pulmonary embolism.

The LV, on the other hand, is accustomed to high afterload and can accommodate extreme rises in afterload (e.g., in a hypertensive crisis) but is itself very sensitive to rises in preload and decompensates rapidly, e.g., in acute aortic valve insufficiency.

Hence, the RV is volume-*tolerant*, but pressure-*intolerant*.

### 2.2. The Right and Left Ventricles are Inseparably Coupled

Through their shared interventricular septum (IVS) the left and right ventricles are inseparably coupled to one another. With an intact pericardium, rising intracavitary pressures in one chamber will automatically lead to a shift of the IVS into the other chamber. The IVS is an important determinant of RV contractile function [9,10] and hence any pathological shifting or wall motion abnormalities of the IVS will have immediate and negative consequences on RV function. Due to the generally higher intracavitary pressures in the LV compared to the RV, the IVS is normally shifted to the right leaving the LV appearing circular in the short axis and the RV crescent-shaped. With elevated RV pressures the IVS may be pushed over to the left, compressing the LV and impairing LV diastolic function, leading to impaired filling and a drop in stroke volume.

Hence, LV and RV function cannot be examined in isolation and a central goal in the management of RV failure is the restoration of an anatomical position of the IVS, be it through reduction of pulmonary vascular resistance (PVR) or optimization of RV preload.

### 2.3. Cardiorespiratory Interactions and the Importance of Spontaneous Respiration in RV Failure

The impact of positive pressure ventilation on RV and LV function is fundamentally different. This is due to the fact that the RV’s preload is extrathoracic and its afterload is intrathoracic, in contrast to the LV with an intrathoracic preload and extrathoracic afterload.

The pulmonary vasculature is intimately anatomically and physiologically intertwined with pulmonary mechanics. This has the consequence that in positive pressure ventilation, inspiration leads to a general increase in RV afterload and drop in RV preload. This stands in contrast to spontaneous respiration, which is associated with generally lower RV afterload and higher RV preload.

The generally disadvantageous effects of positive pressure ventilation, as well as the U-shaped dependency of pulmonary vascular resistance (PVR) on lung volume with its optimum at functional residual capacity (FRC) imply that patients with RV dysfunction or failure should whenever possible be maintained in spontaneous respiration and if pressure support modes or positive pressure ventilation are employed, intrathoracic pressures should be minimized and tidal volumes optimized with the goal of maintaining FRC.

### 2.4. Right Ventricular Dysfunction—Acute versus Chronic

When discussing RV dysfunction and failure, it is imperative to consider the acuity of the underlying pathophysiology—chronic and acute RV dysfunction differ markedly in terms of prognosis as well as perioperative implications.

**Figure 1:**
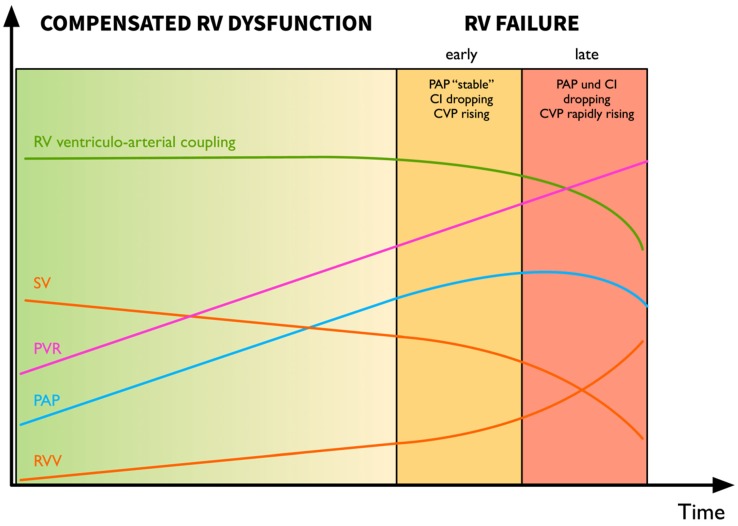
The progression from right ventricular (RV) dysfunction to failure is a continuum marked by progressive RV dilation and increases in heart rate to maintain cardiac index. When the compensatory mechanisms are exhausted, ventriculo-arterial uncoupling occurs with a drop in cardiac index and pulmonary pressures as well as a rise in central venous pressure, late markers of RV failure and imminent cardiovascular demise. Abbreviations: CI, cardiac index; CVP, central venous pressure; PAP, pulmonary artery pressure; PVR, pulmonary vascular resistance; RV, right ventricular; RVV, right ventricular volume; SV, stroke volume. Modified from Haddad et al. [8] Vonk Noordegraaf et al. [11] and Wanner PM & Filipovic M. (Der rechte Ventrikel—das Wichtigste für den Intensivmediziner. In: Eckart, Forst, Briegel, eds.: Intensivmedizin. Kompendium und Repetitorium zur interdisziplinären Weiter- und Fortbildung. ecomed Verlagsgesellschaft AG & Co., Landsberg. 2018) [12].

When examining the clinical trajectory of a patient with *chronic* pulmonary hypertension (PH), one can observe a long phase of compensated RV dysfunction before compensatory mechanisms are exhausted and overt RV failure manifests itself (Figure 1). Pathophysiologically, the RV initially adapts to increased PVR by increasing contractility as well as through hypertrophy. With persistent PH, the RV begins to dilate and increase heart rate to maintain cardiac output. In the final stage of RV dysfunction, ventriculo-arterial uncoupling ensues with reduced cardiac output and RV failure [11].

Preoperatively these patients may clinically show signs of chronic right-sided or biventricular heart failure. Recognizing that any unnecessary surgical procedures should be avoided, these patients generally tolerate a carefully performed general anesthetic relatively well, assuming they still have cardiovascular reserves. The key in these patients is a rigorous preoperative risk stratification, with the goal of avoiding unnecessary procedures and identifying patients at the end of their compensatory mechanisms, in whom general anesthesia is associated with a markedly elevated perioperative risk.

**Figure 2:**
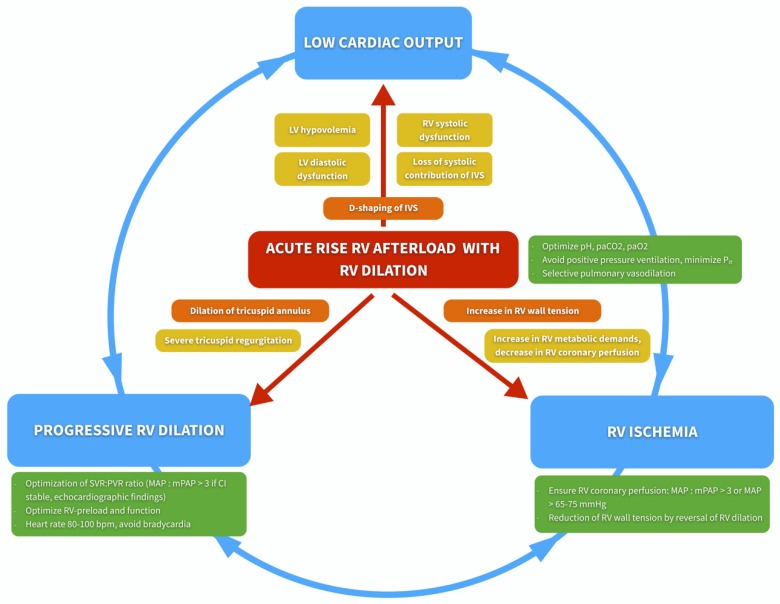
Vicious circle of right ventricular failure. Abbreviations: CI, cardiac index; IVS, interventricular septum; LV, left ventricular; MAP, mean arterial pressure; mPAP, mean pulmonary artery pressure; P_it_, intrathoracic pressure; PVR, pulmonary vascular resistance; RV, right ventricular; SVR, systemic vascular resistance. Modified from Wanner PM & Filipovic M. (Der rechte Ventrikel—das Wichtigste für den Intensivmediziner. In: Eckart, Forst, Briegel, eds.: Intensivmedizin. Kompendium und Repetitorium zur interdisziplinären Weiter- und Fortbildung. ecomed Verlagsgesellschaft AG & Co., Landsberg. 2018) [12].

On the other hand, patients with *acute* RV dysfunction will with high likelihood experience cardiovascular demise if exposed to general anesthesia as the unprepared RV does not have the time to adapt with compensatory mechanisms. What ensues is a sequence of events beginning with RV contractile dysfunction, rapidly progressing to RV failure, culminating in cardiogenic shock and death. On the pathophysiologic level, RV dilation, RV ischemia, and systemic hypotension are central factors perpetuating this vicious circle (Figure 2).

## 3. Challenges in the Preoperative Setting

### 3.1. Pulmonary Hypertension as an Underestimated and Relevant Perioperative Risk Factor

As in all domains of perioperative medicine and anesthesiology, anticipation and avoidance of problems is always superior to treatment of complications. However, in patients with RV dysfunction prevention is paramount.

Known severe pulmonary hypertension is a significant risk factor for perioperative morbidity and mortality [4,5]. In a study of patients with preexisting pulmonary arterial hypertension undergoing in-hospital cardiopulmonary resuscitation [13], not only was successful resuscitation rare, but in 50% of patients relatively minor intercurrent disease (respiratory and gastrointestinal tract infections) had preceded the cardiac arrest, illustrating how fragile this patient population is. Although in some patients the diagnosis of PH will have been made preoperatively, in many it is up to the anesthesiologist to actively search for conditions predisposing to PH (e.g., severe cardiac disease, particularly severe left heart failure and hemodynamically relevant valve lesions; severe obstructive & restrictive pulmonary disease; severe obesity hypoventilation syndrome) and to be alert to signs of right-sided or biventricular heart failure (exercise intolerance, peripheral edema, dyspnea, angina pectoris, etc.). The clinical exam has been shown to be insensitive for the detection of PH [14], but can still yield valuable clues when evaluating patients preoperatively.

### 3.2. Diagnostic Strategy for the Workup of Suspected Pulmonary Hypertension

The *2015 ESC Guidelines for the diagnosis and treatment of pulmonary hypertension* outline an evidence-based diagnostic algorithm to guide workup of clinically suspected PH [15]. Transthoracic echocardiography (TTE) assumes a central role in the evaluation of the RV and is recommended as a first-line screening examination for PH (class I recommendation). Generally, patients with signs of severe PH/RV dysfunction or those with presumed etiologies other than left heart or lung disease should be referred to a PH center for further workup and management.

In all patients with high echocardiographic probability of PH, right heart catheter (RHC) is recommended (class I) [15]. When risk factors or associated conditions for pulmonary artery hypertension (PAH) or chronic thromboembolic pulmonary hypertension (CTEPH) are present together with an intermediate echocardiographic probability of PH, RHC should be considered (class IIa) [15].

Many other diagnostic adjuncts may be used depending on the clinical situation. One interesting imaging modality is cardiovascular magnetic resonance imaging (MRI), as it allows precise non-invasive measurement of RV size, morphology, mass and function, as well as detection of myocardial inflammation and fibrosis, and is by many considered the gold standard for these clinical questions [16]. Additionally, with its high spatial and temporal resolution cardiovascular MRI may enable a deeper understanding of the pathophysiologic underpinnings of PH and RV dysfunction [17].

### 3.3. Risk Stratification—The Balance Between Operative Indication and Perioperative Risk

The single most important question is that of the operative indication. The simplest way to avoid a perioperative adverse outcome is to not expose the patient to the physiologic stress of an operation and anesthetic in the first place. This is also reflected in the *2014 ESC/ESA Guidelines on non-cardiac surgery* which recommend that surgical interventions in patients with pulmonary artery hypertension be avoided unless absolutely necessary [18]. Identification of risk factors for perioperative RV failure must give rise to a multidisciplinary discussion considering the risks and benefits of surgery in the greater context of the patient’s comorbidities, ideally in a center with expertise in the management of pulmonary hypertension [18].

#### 3.3.1. The Role of Biomarkers

Preoperative risk stratification in noncardiac surgery has undergone a paradigm change in the last years with the use of natriuretic peptides now becoming a central element in evidence-based guidelines [19]. This evolution finds its basis in a large body of evidence demonstrating a strong association between elevated preoperative B-type natriuretic peptide (BNP) levels and the occurrence of major adverse cardiac events (MACE) [20].

There is strong clinical evidence for the use of natriuretic peptides in risk stratification of not only heart failure in general, but of RV dysfunction in particular. It has been shown that BNP and N-terminal prohormone of BNP (NT-proBNP) can be used not only for prognostication in patients with chronic primary PH [21,22,23], but also in states of acute RV dysfunction such as acute heart failure in primary PH [24,25] and acute pulmonary embolism [25,26]. Particularly attractive in the perioperative setting are BNP’s respectively NT-proBNP’s high sensitivities, high negative predictive values and low negative likelihood ratios with regard to MACE, making them excellent preoperative screening tools [27].

#### 3.3.2. The Role of Echocardiography

A question that often arises is that of the indication for preoperative echocardiography for risk stratification. Although various echocardiographic parameters have been associated with post-operative adverse outcomes [28,29], in a large retrospective cohort study of 264,823 patients undergoing major noncardiac surgery preoperative echocardiography (performed in 40,084 patients) was not associated with improved survival or reduced hospital stay [30]. Further, compared to NT-proBNP, preoperative echocardiographic parameters (LV ejection fraction, regional wall motion abnormalities, left atrial volume index, diastolic dysfunction) showed inferior predictive power for MACE following major noncardiac surgery and—unlike NT-proBNP—did not add incremental predictive value to the Revised Cardiac Risk Index [29]. These findings form the basis for the strong recommendation of the *Canadian Cardiovascular Society Guidelines on Perioperative Cardiac Risk Assessment and Management of Patients Who Undergo Noncardiac Surgery* against routinely performing resting echocardiography to enhance perioperative risk estimation [19]. However, these guidelines foresee three exceptions: clinical suspicion of undiagnosed severe pulmonary hypertension, of severe obstructive intracardiac abnormalities (aortic stenosis, hypertrophic obstructive cardiomyopathy, mitral stenosis) or of cardiomyopathy. Hence, in patients with known or suspected PH, it is our opinion that a preoperative echocardiogram is indicated, as these patients represent a particularly high-risk cohort and preoperative echocardiography with a focus on RV function may enable more effective risk stratification and optimization [31].

As mentioned above, the progression of RV dysfunction to failure in chronic PH is a continuum. The challenge preoperatively is to determine at what point in this natural disease course the patient is currently located. Unfortunately, there is a paucity of evidence to guide clinicians in answering this key question. As always, preoperative risk stratification should always be multimodal, based on clinical indicators, biomarkers, echocardiographic parameters and, when indicated, further diagnostic adjuncts.

A promising echocardiographic parameter that could help to answer the question as to how many cardiopulmonary reserves have been exhausted is the ratio of tricuspid annular plane systolic excursion to pulmonary artery systolic pressure (TAPSE/PASP). A growing body of evidence suggests that this ratio provides more information than either parameter considered alone and that it reflects the extent of ventriculo-arterial uncoupling—a central event in the progression from RV dysfunction to failure—and that the TAPSE/PASP ratio may help stratify risk in patients and is associated with relevant clinical adverse outcomes [32,33,34,35,36].

### 3.4. Formulating a Multidisciplinary Perioperative Treatment Strategy

Important elements of a perioperative treatment strategy are optimization of the surgical and anesthetic plans to minimize the physiologic insult, preoperative optimization of cardiovascular medications (particularly of pulmonary vasodilators) and planning of the post-operative phase with attention to the exit strategy should complications occur.

A central surgical factor is the invasiveness of the operation (minor vs. moderate-major noncardiac surgery), which determines the duration of surgery, the risk of large fluid shifts and the extent of postoperative systemic inflammatory response to expect. Traditionally laparoscopic surgery has been viewed as universally less invasive than open surgery. However, from a physiologic standpoint the use of pneumoperitoneum, with the associated hypercarbia and elevated intraabdominal/intrathoracic pressures may cause larger physiologic compromise in a patient with severe pulmonary hypertension than an open procedure [37]. Hence, the trauma of surgery must be weighed against the physiologic sequelae of laparoscopy.

A key anesthetic factor is the *avoidance of general anesthesia* – whenever possible a regional or neuraxial anesthetic technique should be chosen, as is reflected by the class IIa recommendation for epidural over general anesthesia for elective surgery in the *2015 ESC Guidelines for the diagnosis and treatment of pulmonary hypertension* [15]. When using neuraxial techniques, care should be given to avoid sudden drops in preload or afterload. The use of a combined spinal-epidural anesthesia (CSEA) with proactively started vasopressors can often provide hemodynamic stability, excellent patient comfort and favorable operating conditions devoid of time constraints [38,39,40]. Important in addition to preventing abrupt changes in cardiac loading conditions is the avoidance of sedation during regional or neuraxial anesthesia due to the risk of hypoventilation, hypercarbia and a rise in PVR, precipitating RV failure.

Lastly, in high-risk patients in whom high-risk surgery with general anesthesia is deemed necessary, a preoperative discussion with the patient regarding operative risk, any advance directives and the extent of medically sensible postoperative support should take place.

## 4. Challenges in the Intraoperative and Postoperative Settings

### 4.1. Prevention of Right Ventricular Failure

It is imperative to avoid rises in pulmonary vascular resistance, drops in systemic blood pressure with RV hypoperfusion and derangements of volemia.

Pulmonary vascular resistance, as outlined above, is intricately linked to respiratory mechanics and whenever possible, the institution of invasive, positive pressure ventilation should be avoided. When intubation is necessary, spontaneous respiration with a minimal amount of pressure support should be considered. If positive pressure ventilation is necessary, intrathoracic pressures and tidal volumes should be minimized. Contrary to popular belief, positive end-expiratory pressure (PEEP) is not contraindicated in RV dysfunction. As long as PEEP contributes to further pulmonary recruitment with a reduction in hypoxic pulmonary vasoconstriction, an improvement in RV function is to be expected. Although the PEEP level will be individual for each patient, typically a level of 5–8 cmH_2_O may be adequate, depending on the baseline pulmonary pathology.

Systemic hypotension must at all costs be avoided as RV ischemia is a potent initiator and perpetuator of RV failure. A hemodynamically very vulnerable phase in the perioperative care of a patient with severe pulmonary hypertension is anesthetic induction. Rapid changes in preload, afterload and contractility must be avoided.

Volemia should be granted particular attention intraoperatively as patients with RV dysfunction are preload-dependent, but also tolerate hypervolemia poorly with rapidly ensuing RV dilation and failure.

### 4.2. Recognition of Right Ventricular Failure

#### 4.2.1. Choice of Diagnostic Modality

In this review we discuss the most important diagnostic modalities in RV failure, to a certain extent taking for granted the diagnosis. However, in real life one is often presented with a rapidly declining patient and multiple open differentials. In undifferentiated perioperative shock, the primary goal is to rapidly rule-out all plausible causes of shock and center in on a presumptive mechanism driving hemodynamic instability. In this context, point-of-care ultrasound (POCUS) and focused echocardiography can rapidly provide the clinician with essential information not only for differentiating the shock state, but also for guiding therapy in real-time. Hence, the primary diagnostic tool in *any* hemodynamically unstable patient in the perioperative setting should be focused transthoracic or transesophageal echocardiography supplemented with additional sonographic examinations as indicated clinically.

In the case of RV failure, once the diagnosis has been made and initial life-saving therapy initiated, a more granular diagnostic approach can be chosen with escalation of invasive monitoring. However, more information does not always translate to clearer picture—one must always be cognizant to interpret diagnostic findings in the clinical context of the individual patient and to always base therapeutic decisions on the synthesis of all available information.

#### 4.2.2. Echocardiography

There are various protocols for rapid echocardiographic/sonographic assessment of – particularly undifferentiated—shock, e.g., the RUSH-exam [41], which the reader is encouraged to consult. In this review we focus on the highest yield elements of our own personal echocardiographic approach to evaluation of the right ventricle. However, the importance of a holistic exam ruling out *all* potential life-threatening causes of shock cannot be overstated. More important than the choice of any specific protocol is systematic consideration of all relevant differentials for shock during *every* exam. For an in-depth discussion of right ventricular echocardiography, the reader is referred to an excellent review by Noordegraaf et al. [42], the *2010 Guidelines for the Echocardiographic Assessment of the Right Heart in Adults* [43] and the *2015 Recommendations for Cardiac Chamber Quantification by Echocardiography in Adults of the European Society of Cardiology* [44].

In our echocardiographic exam, we search for three key findings of RV failure:

- Signs of RV dilation: D-shaping, increased RV:LV ratio, tricuspid regurgitation
- Signs of impaired RV systolic function: reduced tricuspid annular plane systolic excursion (TAPSE)
- Signs of elevated RV preload (plethoric inferior vena cava [IVC])

**Figure 3:**
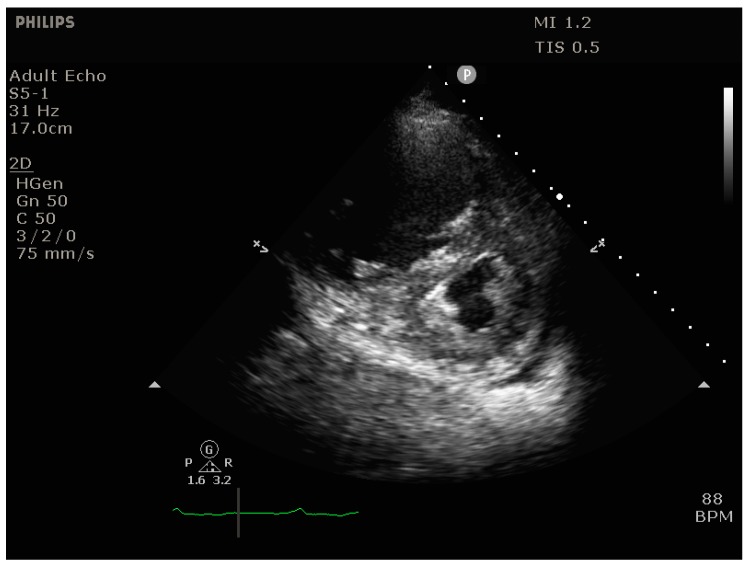
Transthoracic echocardiogram (parasternal short axis view) showing massive D-shaping of the interventricular septum.

**Figure 4:**
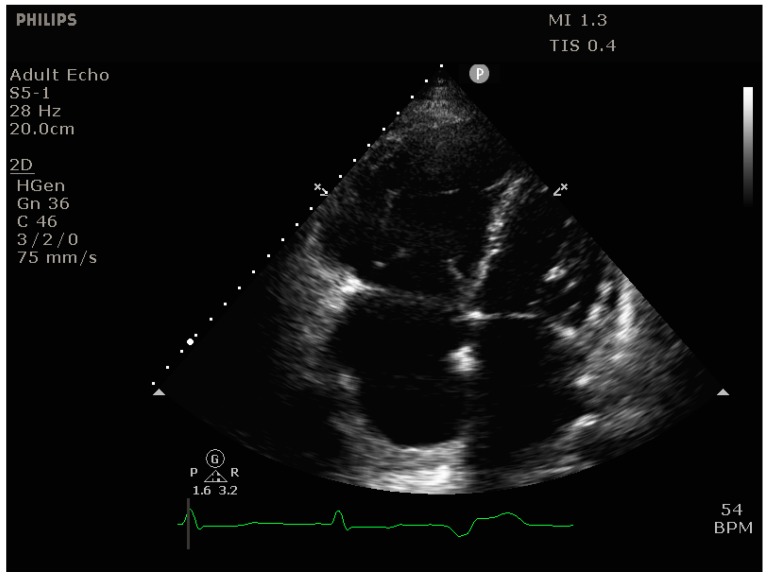
Transthoracic echocardiogram (apical 4-chamber view) showing massive right ventricular dilation (RV diameter > LV diameter).

**RV dilation** is most easily visualized in the parasternal short-axis view (transesophageal echocardiography [TEE]: transgastric short axis view), where it manifests itself as bulging of the interventricular septum into the LV (“D-shaping”), (Figure 3 and Figure 4*)*. It is useful to note whether D-shaping is only present in diastole or in all phases of the cardiac cycle, as this can provide clues to the etiology of RV failure (systolic & diastolic D-shaping primarily in RV pressure overload, isolated diastolic D-shaping appearing as an IVS flopping back and forth primarily in RV volume overload.

Another sign of RV dilation is an increased RV:LV diameter ratio in the apical 4-chamber view (TEE: mid-esophageal 4-chamber view), with RV:LV > 0.6 indicating RV dilation and RV:LV > 1 indicating severe RV dilation. 

**Figure 5:**
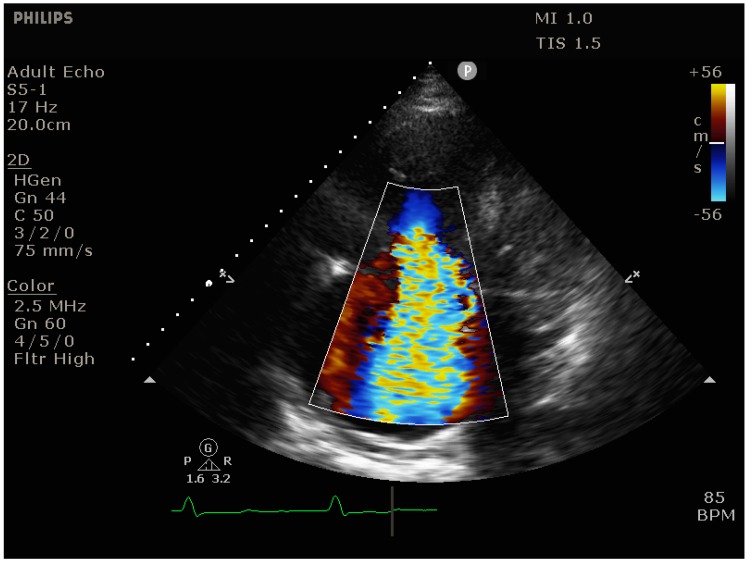
Transthoracic echocardiogram (apical 4-chamber view) showing severe tricuspid regurgitation due to massive RV dilation.

Lastly, in severe RV dilation the tricuspid annulus gets passively stretched leading to often severe functional tricuspid regurgitation (TR), (Figure 5). Much less important than precise quantification of the TR is recognition of severe TR as an indication of significant annular dilatation and/or severe PH.

*Qualitative* indicators of severe TR are the presence of a central TR jet filling the right atrium or a wall-hugging eccentric TR jet (Coanda effect), which can be rapidly visualized with color flow doppler (CFD) in the apical 4-chamber view (TEE: mid-esophageal 4-chamber view).

Two echocardiographic parameters that permit *semi-quantitative* staging of TR are: [45].

- Vena contracta (VC) width: The width of the regurgitant jet is measured as it leaves the regurgitant orifice. It is best imaged perpendicular to the valve plane, e.g., in the apical 4-chamber view, using CFD with a Nyquist limit of 50–60 cm/s. The VC width corresponds to the width of the narrowest portion of the jet (the “neck), with values > 7 mm suggestive of severe TR.
- Proximal isovelocity surface area (PISA): [46] With significant TR, there is a flow acceleration proximal to the regurgitant valve orifice. This proximal flow convergence occurs along concentric hemispheres and can be visualized using CFD with an adequately set Nyquist limit (15–40 cm/s) to maximize delineation of the flow acceleration. The radius of the PISA correlates with TR severity, with values > 9 mm suggestive of severe TR.

It is important to note that the severity of a TR jet can be underestimated if there is near-equalization of right atrial and right ventricular pressures, e.g., in the case of massive TR due to a wide, noncoapting tricuspid valve or due to chronically elevated right atrial pressures [45,47].

When TR is present, the speed of the jet can be used to estimate the systolic pulmonary artery pressure (sPAP) using the Bernoulli equation. Although echocardiographic sPAP estimations are useful in patients with chronic PH in the steady state, they should be interpreted with caution in RV failure as they can be inaccurate in this setting. [48] Hence, if pulmonary vascular pressures are deemed necessary to guide further treatment, invasive monitoring should be escalated and a pulmonary artery catheter (PAC) inserted.

**Figure 6:**
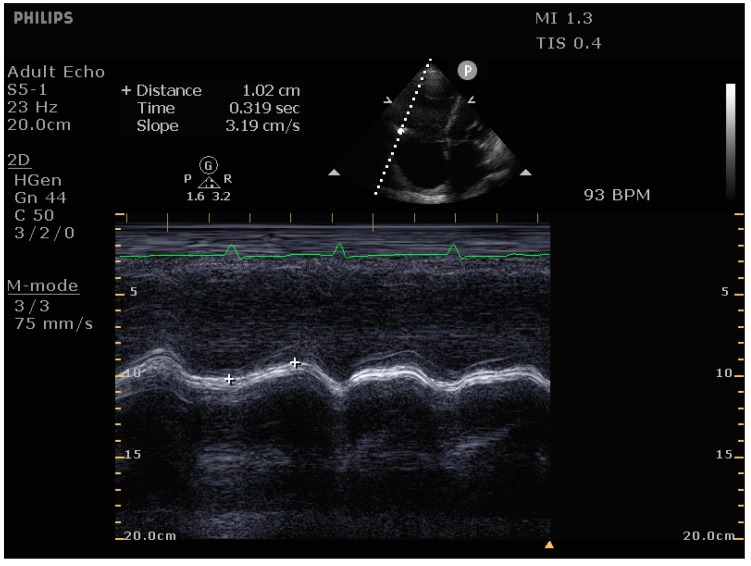
Measurement of tricuspid annular plane systolic excursion (TAPSE) as a measure of RV systolic function. Values < 17 mm are considered pathological (here 10 mm).

Due to the RV’s primarily longitudinal contraction, the TAPSE is a good estimate of RV systolic function. [49] This is best measured in the apical 4-chamber view (TEE: mid-esophageal 4-chamber view) in M-mode while assuring optimal alignment of the lateral tricuspid annulus movement with the measurement beam (Figure 6). TAPSE values below 17 mm are considered pathological, indicating impaired RV systolic function. Importantly, in the setting of severe TR, measures of RV longitudinal function such as TAPSE may overestimate RV systolic function due to the decreased RV afterload and ensuing significant regurgitant volume.

Evaluation of the IVC is best accomplished in the subcostal 4-chamber view using M-Mode to examine the temporal change of IVC diameter. One must note that although sensitive for RV failure, this sign is not specific, especially in mechanically ventilated patients. Hence, a small IVC with respiratory variability should lead to questioning of the working diagnosis of RV failure.

It must be stressed that no single echocardiographic finding or measurement should be used in isolation as a basis for treatment decisions. It is vital to integrate all collected echocardiographic information with the clinical findings. Lastly, the clinician should try to develop a sense for recognizing severely impaired RV function and severe RV dilation (“eyeballing”), as this can not only speed up the diagnostic process [50,51], but also aid in interpreting further echocardiographic findings. However, the one must be cognizant of the significant learning curve required to do this accurately and reproducibly [52].

#### 4.2.3. Pulmonary Artery Catheter

Over the years, the use of the pulmonary artery catheter (PAC, Swan-Ganz) has been increasingly questioned [53,54,55]. Its major perioperative role is in the management of complex cardiac surgical patients. However, it remains an important tool in noncardiac surgery in the management of patients with severe RV failure. Through the direct measurement of pulmonary artery pressures (PAP) and the determination of cardiac output by thermodilution, the hemodynamic response to therapy can be followed in real-time, enabling rational optimization of therapy. An exhaustive discussion of pulmonary hemodynamics is beyond the scope of this review. However, we will emphasize core points in the use of the PAC in RV failure. For the reader seeking more information on the use of the PAC in PH, we can recommend the concise review by Rosenkranz et al. [56].

When using a PAC, key hemodynamic parameters are:

- Mean pulmonary artery pressure (mPAP)
- Cardiac output (CO)
- Pulmonary artery wedge pressure (PAWP), also known as pulmonary artery occlusion pressure (PAOP)

The mPAP is important both in the staging of the severity of pulmonary hypertension as well as in the determination of its etiology [15]. Recently the mPAP cutoff for the definition of PH was lowered from 25 to 20 mmHg, reflecting a transition from an arbitrary definition to one based on population data [57]. It is important to always interpret an elevated mPAP together with other hemodynamic variables such as the cardiac output and PAWP.

One can differentiate three phenotypes of pulmonary hypertension (PH):

- Pre-capillary PH: mPAP > 20 mmHg, PAWP ≤ 15 mmHg, PVR ≥ 3 Woods units
  Typical causes: pulmonary artery hypertension (PAH), lung disease and/or hypoxia, pulmonary artery obstruction
- Isolated post-capillary PH: mPAP > 20 mmHg, PAWP > 15 mmHg, PVR < 3 Woods units
  Typical causes: left heart disease
- Combined pre- and post-capillary PH: mPAP > 20 mmHg, PAWP > 15 mmHg, PVR ≥ 3 Woods units
  Typical causes: left heart disease as well as unclear and/or multifactorial mechanisms

This distinction is of importance, since the required therapeutic interventions differ between groups. *Pre-capillary* PH is generally due to pulmonary parenchymal or vascular disease and hence selective pulmonary vasodilation will form an important therapeutic pillar. *Post-capillary PH,* on the other hand, is generally due to decompensated left heart disease with elevated filling pressures and hence the most important therapeutic intervention will be treatment of left-sided heart failure.

#### 4.2.4. Recognition of the Decompensating Right Ventricle—Pressure Does Not Equal Flow

When interpreting pulmonary artery pressures, it is vital to always consider how cardiac output is trending in comparison to the PAP. The combination of dropping pulmonary artery pressures and sinking cardiac output is a harbinger of impending RV failure. As is illustrated by the equation PAP = PVR * CO, both reductions in PVR and/or CO will lead to a reduction in PAP—pressure does not equal flow. Hence, only when a drop in PAP is accompanied by an increase in CO can one assume that therapeutic interventions have functioned and the patient is improving.

When PAC monitoring is not available, a central venous line with continuous central venous pressure (CVP) monitoring can also provide an indication of progressive RV decompensation. Hence, an elevated and rising CVP must always lead to further examinations (ideally echocardiography) to rule out impending RV failure or other causes of obstructive shock (e.g., pericardial tamponade, tension pneumothorax).

### 4.3. Management of RV Failure

#### 4.3.1. Immediate Measures to Break out of the Spiral of RV Failure

As illustrated above, once RV failure has begun, a vicious circle of systemic hypotension with RV ischemia and RV dilation is set in motion. Unless immediately terminated, this vicious circle will lead to a rapid hemodynamic decline.

The most important intervention in the hypotensive patient with RV failure is to immediately correct systemic hypotension through the administration of push-dose vasopressors (e.g., norepinephrine or epinephrine). In parallel, running vasopressor infusions should be increased, taking into account a prolonged circulatory time. A potentially attractive vasopressor once systemic hypotension has been reversed is vasopressin, as it carries the theoretical advantage that its pressor effect is selective for the systemic circulation, unlike norepinephrine and epinephrine which can both cause a rise in PVR when administered in high doses. 

There are sparse data on the ideal target blood pressure in RV dysfunction—each patient will require individualization based on their clinical response to treatment. However, guaranteeing a mean arterial pressure (MAP) > 65–75 mmHg would seem prudent.

Once systemic hypotension has been reversed, the next treatment step with persistent RV dysfunction is to eliminate reversible causes of increased PVR:

- Hypoxia & hypercarbia: Careful titration of PEEP can prove helpful in optimizing gas exchange and can lead to a reduction in PVR. In non-intubated patients, careful application of non-invasive ventilation (NIV) with titration of PEEP to gas exchange and hemodynamics can help delay intubation and enable the treatment of underlying causes. Another potentially promising alternative to intubation is the use of high-flow nasal cannula (HFNC) which enables optimization of oxygenation with often superior patient tolerance and less cardiovascular sequelae compared to NIV [58].
- Increased intrathoracic pressure: in the mechanically ventilated patient, care must be taken to minimize intrathoracic pressures (plateau < 27 mmHg [59]) and tidal volumes.
- Increased sympathetic tone (avoid stress through adequate analgosedation, normothermia)

With persisting RV failure, the next step is to establish selective pulmonary vasodilation and to escalate monitoring with the addition of a pulmonary artery catheter (see below). Pharmacologic options include inhaled nitric oxide [60], inhaled prostaglandins (e.g., iloprost, common practice but *off-label* use) [60,61] or inhaled phosphodiesterase III inhibitors (e.g., milrinone [62], *off-label* use). Whenever possible, in the acute phase of RV failure selective pulmonary vasodilation must be chosen above systemic pulmonary vasodilators, as the latter can cause significant systemic hypotension, worsening RV failure [63]. Once hemodynamic stability has been achieved (in the subacute to chronic phases), systemic pulmonary vasodilators such as sildenafil play an important role in the further optimization of patients with PH.

#### 4.3.2. Treatment of the Underlying Cause

**Figure 7:**
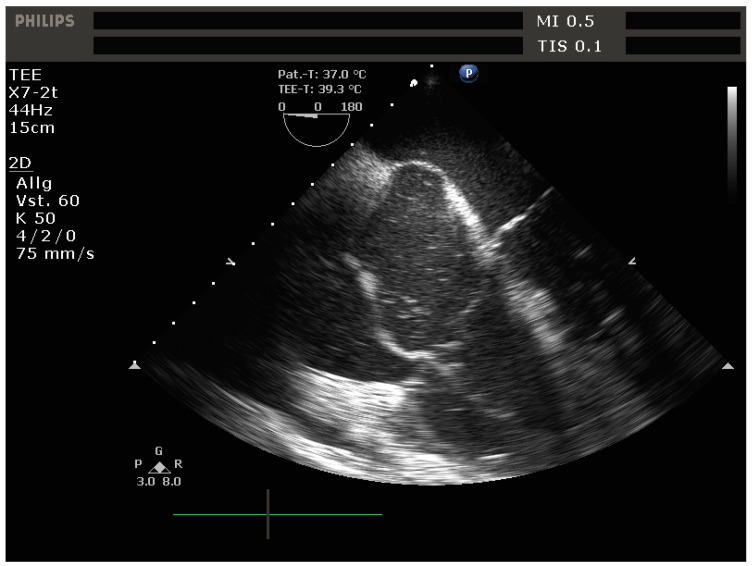
Transesophageal echocardiogram (midesophageal 4-chamber view) showing severe dilation of the right-sided cardiac chambers, bowing of the interatrial septum into the LV due to elevated right atrial pressures and a hyperechogenic structure in the RA and RV, in this case a clot in transit.

In parallel to the above-mentioned interventions, treatment of the underlying cause of RV failure should be undertaken. In the perioperative setting it helps to primarily focus on and rule out the most common etiologies of RV failure:

- Progression of pre-existing pulmonary hypertension: removal of exacerbating factors, selective pulmonary vasodilation
- Embolic events: pulmonary embolism (Figure 7), fat embolism, bone cement embolism, amniotic fluid embolism, air/gas embolism, etc: thrombolysis if applicable, supportive measures, selective pulmonary vasodilation
- LV backwards failure due to large myocardial infarction and/or severe mitral regurgitation: recompensation of heart failure, mechanical circulatory support, surgical options
- RV myocardial infarction: coronary revascularization if indicated (ST-elevation myocardial infarction [STEMI] and/or cardiogenic shock), supportive measures, mechanical circulatory support

Perioperative high-risk pulmonary embolism poses a unique problem in that many of the recommended therapeutic options (thrombolysis, anticoagulation) may not be possible in the postoperative phase due to the elevated risk of bleeding complications. Such cases require individualized, interdisciplinary discussion between anesthesiologists, intensivists and surgeons, balancing the risk of further hemodynamic deterioration with the risk of bleeding. The key surgical question is whether bleeding would be expected to occur into a life-threatening space or to a life-threatening extent, or whether increased postoperative bleeding could be bridged with transfusions. A pragmatic, yet *not* evidence-based approach is starting the patient as soon as surgically feasible on a moderate dose of unfractionated heparin (approximately 10–15 kIE/24 h) without bolus and cautiously yet steadily increasing the dose while monitoring for bleeding complications, if necessary pausing or reducing the infusion. What must at all costs be avoided is reversal of anticoagulation using procoagulants, since this increases the risk of further thromboembolic complications dramatically and often leads to a pendulum effect, overshooting in one and then the other direction. In the case of refractory shock, simple anticoagulation will not suffice and catheter-based thrombolysis or reduced-dose systemic thrombolysis can be considered and, if compatible with the patient’s general prognosis, early initiation of mechanical circulatory support should be discussed.

#### 4.3.3. Management of Volemia in RV failure

In RV failure, a differentiated approach to fluid therapy is necessary as many patients will already find themselves on the descending portion of the Frank-Starling curve with further fluid administration leading to progressive RV dilation, impaired RV contractility, reduced LV filling and a further drop in cardiac output. On the other hand, patients in RV failure are often preload-dependent, with over-aggressive fluid removal also leading to clinical deterioration. The key is the use of passive leg raising or anti-Trendelenburg positioning under real-time cardiac output or echocardiographic monitoring to help predict the patient’s response to fluid administration or removal. In the case of worsened hemodynamics, the positioning can be easily reversed. This technique is of particular value in RV failure as typically employed measures of volume responsiveness such as stroke volume variation or pulse pressure variation can be falsely high [64].

When the decision is made to administer fluids, small aliquots should be given at a time (ca. 100–250 mL, the “mini-fluid challenge” [65]). On the other hand, when one decides to remove fluid, interventions easily stopped (e.g., fluid removal via continuous renal replacement therapy) or with short half-lives (e.g., nitroglycerin) should be chosen before longer acting medications (e.g., diuretics) are given.

#### 4.3.4. Support of Inotropy in RV Failure

In the non-hypertrophied RV, the lower muscle mass translates to a smaller increase in contractility with administration of inotropes than would be expected for the LV. However, in refractory RV failure the administration of inotropes is often necessary to maintain cardiac output and systemic perfusion.

First-line medications that can be used to rapidly stabilize hemodynamics are the beta-mimetics dobutamine or epinephrine. When using dobutamine, care must be given to increase the norepinephrine infusion rate to compensate for the reduction in systemic vascular resistance (SVR) due to beta-2-agonism. The pure alpha-1-agonist phenylephrine should be avoided due to the possibility of an increase in PVR [66,67].

Once hemodynamics have been stabilized, in patients with persisting severe RV contractile dysfunction consideration can be given to the use of long-acting inodilators such as the phosphodiesterase 3 inhibitor milrinone [68] or the calcium sensitizer levosimendan [69], with both agents having been shown to improve hemodynamics. If either agent is administered, care must be taken due to their systemic vasodilatory effects and under no circumstances should a bolus be given.

Importantly, it must be noted that there is a paucity of high-quality data supporting the use of *any* of the mentioned inotropes or inodilators [63]. Dobutamine and milrinone both augment RV systolic function in right ventricular failure [68]. However, there is a lack of robust outcome data. Evidence for the use of epinephrine in RV failure is equally sparse, however, its use in cardiogenic shock has come under scrutiny as it was recently shown in a large meta-analysis to be associated with increased short-term mortality [70]. One meta-analysis found significant hemodynamic improvements with levosimendan [71], perhaps suggesting an indication in selected patients, but here too there is a paucity of longer-term outcome data.

Hence, the author’s practice and recommendation is to remain conservative in the use of inotropes in RV failure and, if deemed necessary, to use the lowest possible dose sufficient to sustain systemic perfusion. In patients with refractory shock and high inotrope requirements, consideration should be given to early escalation to mechanical circulatory support, provided this is compatible with the patient’s prognosis.

#### 4.3.5. Arrhythmias in RV Failure

The whole spectrum of arrhythmias from supraventricular to ventricular and tachyarrhythmias to bradyarrhythmias can be seen in severe RV failure. Principally, an important hemodynamic goal in RV failure is maintenance of a normocardic sinus rhythm. When severe TR is present, bradycardia should be avoided and slightly normo-tachycardic heart rates (80–100 bpm) can help reduce regurgitation fraction.

Common tachyarrhythmias are sinus tachycardia, atrial fibrillation and atrial flutter. In most cases, these tachyarrhythmias are the indication of demand tachycardia and maximal sympathetic tone due to impaired LV filling and falling stroke volume. The most important intervention is treatment of RV failure and of the underlying cause. Under no circumstance should rate control with beta blockers or calcium channel blockers be attempted, as this will precipitate cardiovascular collapse. Cautious rate or rhythm control with amiodarone or ibutilide can be attempted. In the case of rapid hemodynamic deterioration linked to rapid atrial fibrillation or flutter, an attempt at electrical cardioversion can be made. However, often such attempts remain futile until the underlying cause of the arrhythmias has been reversed. In awake, non-intubated patients the risks associated with analgosedation in a cardiopulmonary marginal state must be considered. The ideal medication in this context is ketamine titrated intravenously in 5–10 mg aliquots to effect. Concerns as to the sympathomimetic effects of ketamine are not justified in these doses in patients with maximal sympathetic tone and depleted catecholamine stores [72].

Bradyarrhythmias in the context of RV failure are an ominous sign and are generally either the indication of a preterminal state in the spiral of RV failure or of severe damage to the conduction system due to an RV infarct. The treatment approach focuses on immediate reversal of bradycardia, guaranteeing adequate systemic blood pressures, lowering PVR and treating the underlying cause of RV failure. As a temporizing measure epinephrine can be administered in 25–100 mcg aliquots to support heart rate and systemic blood pressure. Transcutaneous pacing should be immediately established in refractory bradycardia followed by insertion of a transvenous pacemaker, anticipating technical difficulties due to pulmonary hypertension and the possibility for malignant arrhythmias.

#### 4.3.6. Anesthetic Induction in the Patient with Right Ventricular Failure

The risk of induction and intubation of a patient with RV failure cannot be overstated. The potential for hemodynamic collapse and cardiac arrest is real. Before proceeding, one must assure that there is indeed no alternative to intubation for improving gas exchange (e.g., high-flow nasal cannula, non-invasive ventilation) and one must have a backup plan in place for the case of hemodynamic demise. Obviously, in the case of severe obtundation or other hard indications for securing the airway, one must proceed with endotracheal intubation.

Patients with RV dysfunction profit from a slow, hemodynamically stable anesthetic induction. Invasive blood pressure monitoring is mandatory. The higher the expected probability of peri-induction hemodynamic instability, the lower the threshold should be to establish central venous access. In the shocked patient, rapid vascular access can be gained through simultaneous femoral arterial and central venous cannulation. Vasoactive medication infusions (e.g., norepinephrine) should be running prior to induction targeting a high-normal blood pressure and push-dose vasopressors (e.g., norepinephrine and/or epinephrine) should be ready. Hemodynamically neutral agents such as ketamine or etomidate should be used. It must be noted that *all* anesthetic agents, including ketamine [73], have the potential for hemodynamic compromise and hence should always be titrated to effect and never given as a large bolus. This is the reason that rapid sequence induction (RSI) should at all costs be avoided in patients with RV failure. The rationale for RSI is avoidance of regurgitation and pulmonary aspiration. However, the risks and benefits of this approach in the face of a very high likelihood of hemodynamic catastrophe must be considered. One option in high-risk patients (e.g., acute abdomen) is pre-induction gastric decompression followed by a delayed-sequence intubation with anesthetics and vasopressors titrated to effect with gentle ventilation (ideally using the respirator to guarantee low inspiratory pressures) during the apneic period to avoid hypercapnia and hypoxia. In patients receiving muscle relaxants to facilitate intubation, hemodynamic compromise may only manifest itself once positive pressure ventilation is initiated.

Another option, especially outside of the operating room when anesthesia and muscle relaxation are not required, is an awake intubation (e.g., awake fiberoptic). Such a scenario could be a patient going to interventional radiology for catheter-directed thrombolysis (CDT) of a high-risk pulmonary embolism whose mental status and metabolic situation are declining due to the shock state and who needs to be bridged for the duration of the intervention. This approach carries the benefit that with a sufficient endotracheal tube size and adequate analgosedation, spontaneous ventilation can be preserved while ensuring airway protection, which is desirable in the most hemodynamically unstable of patients.

#### 4.3.7. Rescue Strategies for Refractory RV Failure

Some patients will not be able to be stabilized despite institution of all these measures. In a selected patient population (those with foreseeably *reversible* causes of RV failure who have not yet sustained irreversible end-organ damage) mechanical circulatory support (MCS) [74] may be indicated. Depending on institutional practices, veno-arterial extracorporeal membrane oxygenation (VA-ECMO) +/− LV-Impella or an RV-Impella [75,76] may be therapeutic options. The *2019 ESC guidelines on acute pulmonary embolism* make a class IIb recommendation for consideration of VA-ECMO combined with either catheter-directed thrombolysis (CDT) or surgical embolectomy in patients with refractory circulatory collapse or cardiac arrest [77]. The use of intra-aortic balloon counterpulsation (IABP) in cardiogenic shock is controversial [78]. Although there is a strong physiologic rationale for use of IABP to augment LV and RV coronary perfusion [79], there is a paucity of data demonstrating its efficacy in RV failure and particularly in biventricular failure an escalation of MCS is often necessary [80].

## 5. Conclusions

Right ventricular failure is an underdiagnosed, common cause of perioperative hemodynamic instability. Its many possible etiologies converge into a vicious circle that, once established, rapidly leads to cardiovascular collapse. Echocardiography plays a central role in the diagnosis as well as in the management of right ventricular failure. Essential therapeutic interventions are the reversal of systemic hypotension, reduction of pulmonary vascular resistance and treatment of the underlying cause of RV failure. The avoidance of intubation and positive pressure ventilation are further important tenets in management of RV failure. Through rapid implementation of these mostly simple interventions, oftentimes the vicious circle can be broken, preventing progression to multi-organ failure and death. Alas, one can only prevent and treat what one has considered—you may forget the RV, but the RV will not forget you.
